# 662. Incidence and Treatment of Liver Abscesses in Ecuador. How Infection Source Control Delay Affects?

**DOI:** 10.1093/ofid/ofad500.725

**Published:** 2023-11-27

**Authors:** Grace Johanna Salazar, Jesús Dawaher, Amy Peralta, Alejandra Borja, Verenice Cóndor, Belén Calucho, Margarita Galarza

**Affiliations:** Hospital Vozandes Quito, Quito, Pichincha, Ecuador; Hospital General Pablo Arturo Suarez, Quito, Pichincha, Ecuador; Centro de Investigación en Enfermedades Infecciosas, México, Distrito Federal, Mexico; Hospital Padre Carolo, Quito, Pichincha, Ecuador; Hospital de Especialidades Axxis, Quito, Pichincha, Ecuador; Hospital General Pablo Arturo Suarez, Quito, Pichincha, Ecuador; Hospital de Especialidades Eugenio Espejo, Quito, Pichincha, Ecuador

## Abstract

**Background:**

Liver abscess is a frequent pathology poorly described in Ecuador. Treatment consists of drainage procedure and antibiotics for a recommended time between 4 to 6 weeks with a low evidence level, however, in other infections it is documented that source control can help decreasing treatment time.

**Methods:**

A multicenter retrospective study was conducted in the public health system hospitals from Ecuador from January 2017 to December 2022. All cases of hospitalized liver abscess patients were included, calculating the incidence with regards to admissions. Demographic, microbiological isolation and treatment data were collected. The frequency of drainage procedure use, time of antibiotic use and length of hospital stay (LOHS) were evaluated. Means and Mann-Whitney U test were used for comparison. A p≤0.05 was considered statistically significant. SPSS version 25 software was used.

**Results:**

With 162 cases, the incidence of the disease was 0.94 per 1000 hospital admissions. Sixty three percent of them were men (106), with an average age of 48 years old (15-91). The underlying causes were described in 33% of the cases. Drainage procedure was performed in 75% of the cases (122), most of them (80%) after the first 72 hours of hospitalization (mean 8.9: 0-35 days), so 84.5% received antibiotics prior to drainage procedure. Etiological microorganism was recovered in 20.9% of the cases (34). Etiology and susceptibility profile can be seen in figure 1. LOHS (19.7 vs. 10.5; p=0.0001; 95% CI 4.6-13.6) and antibiotic use (24.5 vs. 18.3; p=0.003; 95% CI 2.1-9. 9) were longer in patients who had drainage, however, these outcomes varied considerably depending on the time in which the drainage procedure was performed; if it was performed within the first 3 days, LOHS and antibiotic use were significantly lower (25.4 vs 10.6 p=0.0001) (28 vs 20.6: p=0.001; 95% CI 3.7-11.0). Figure 2.

**Figure 1.**
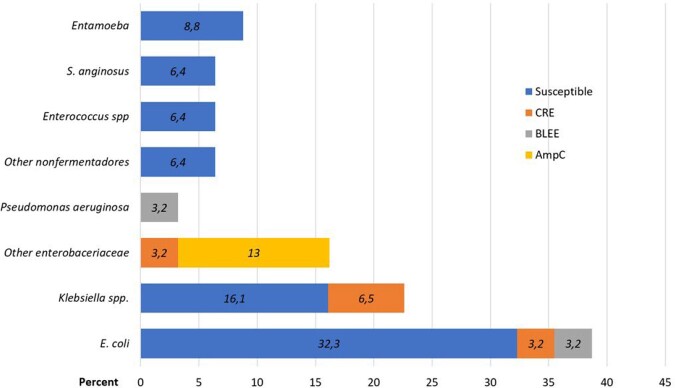
Etiology and susceptibility profile agents of liver abscess in Ecuador. January 2017 - December 2022.

**Figure 2.**
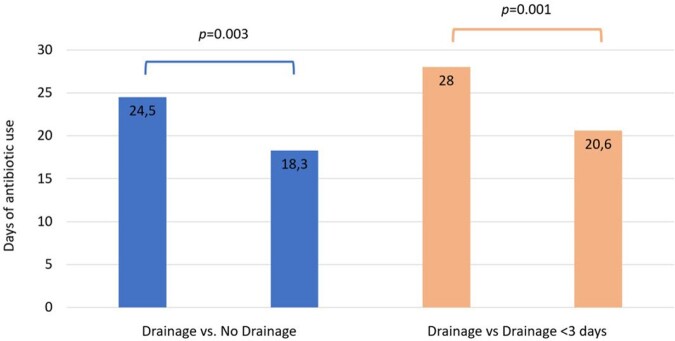
Comparison of antibiotic use days. Global Drainage versus no Drainage and less than 3 days Drainage versus no Drainage.

**Conclusion:**

Our findings show that delaying the drainage procedure for liver abscess treatment decreases microbiological recovery, prolongs LOHS and the use of antibiotics. It is important to identify the reasons why there is no timely intervention in the public health system hospitals in the country to carry out improvement interventions. More studies are needed to optimize the treatment of this pathology.

**Disclosures:**

**All Authors**: No reported disclosures

